# Size-adjusted muscle power and muscle metabolism in patients with cystic fibrosis are equal to healthy controls – a case control study

**DOI:** 10.1186/s12890-019-1039-8

**Published:** 2019-12-30

**Authors:** Katharina Ruf, Meinrad Beer, Herbert Köstler, Andreas Max Weng, Henning Neubauer, Alexander Klein, Kathleen Platek, Kristina Roth, Ralph Beneke, Helge Hebestreit

**Affiliations:** 1 University Children’s Hospital Würzburg, University of Würzburg, Josef-Schneider-Strasse 2, 97080 Würzburg, Germany; 2 Department of Diagnostic and Interventional Radiology, University Hospital Ulm, Ulm, Germany; 3 Department of Diagnostic and Interventional Radiology, University Hospital Würzburg, University of Würzburg, Würzburg, Germany; 4 SRH Poliklinik Radiologie Suhl, Suhl, Germany; 5 Institut für Sportwissenschaft und Motologie, Philipps Universität Marburg, Marburg, Germany

**Keywords:** Cystic fibrosis, Exercise capacity, MRI spectroscopy, Muscle power, Phosphorylation, Lung disease, Muscle function

## Abstract

**Background:**

Skeletal muscle function dysfunction has been reported in patients with cystic fibrosis (CF). Studies so far showed inconclusive data whether reduced exercise capacity is related to intrinsic muscle dysfunction in CF.

**Methods:**

Twenty patients with CF and 23 age-matched controls completed an incremental cardiopulmonary cycling test. Further, a Wingate anaerobic test to assess muscle power was performed. In addition, all participants completed an incremental knee-extension test with ^31^P magnetic resonance spectroscopy to assess muscle metabolism (inorganic phosphate (Pi) and phosphocreatinine (PCr) as well as intracellular pH). In the MRI, muscle cross-sectional area of the *M. quadriceps* (qCSA) was also measured. A subgroup of 15 participants (5 CF, 10 control) additionally completed a continuous high-intensity, high-frequency knee-extension exercise task during ^31^P magnetic resonance spectroscopy to assess muscle metabolism.

**Results:**

Patients with CF showed a reduced exercise capacity in the incremental cardiopulmonary cycling test (VO_2_peak: CF 77.8 ± 16.2%predicted (36.5 ± 7.4 ml/qCSA/min), control 100.6 ± 18.8%predicted (49.1 ± 11.4 ml/qCSA/min); *p* < 0.001), and deficits in anaerobic capacity reflected by the Wingate test (peak power: CF 537 ± 180 W, control 727 ± 186 W; mean power: CF 378 ± 127 W, control 486 ± 126 W; power drop CF 12 ± 5 W, control 8 ± 4 W. all: *p* < 0.001). In the knee-extension task, patients with CF achieved a significantly lower workload (*p* < 0.05). However, in a linear model analysing maximal work load of the incremental knee-extension task and results of the Wingate test, respectively, only muscle size and height, but not disease status (CF or not) contributed to explaining variance. In line with this finding, no differences were found in muscle metabolism reflected by intracellular pH and the ratio of Pi/PCr at submaximal stages and peak exercise measured through MRI spectroscopy.

**Conclusions:**

The lower absolute muscle power in patients with CF compared to controls is exclusively explained by the reduced muscle size in this study. No evidence was found for an intrinsic skeletal muscle dysfunction due to primary alterations of muscle metabolism.

## Background

Cystic Fibrosis (CF) is a rare, life-limiting disease associated with reduced aerobic and anaerobic exercise capacity resulting in poor exercise tolerance [1–4]. Several studies reported reduced muscle function in patients with CF compared to healthy controls leading to constraints in aerobic and anaerobic capacities [5, 6]. In CF, aerobic exercise capacity is related to major clinical consequences such as survival and lung transplantion [7, 8]. Interestingly, both, aerobic and anaerobic exercise capacity are related to quality of life [9]. In addition to pulmonary function and physical activity, muscle function is an important predictor of aerobic capacity [10]. Studies have proven that improving lung function by bronchodilators does not increase peak aerobic capacity [11]; at the same time, patients with CF are able to fatigue peripheral muscles despite ventilatory limitations [12]. Both aspects highlight the importance of peripheral muscle function for exercise capacity.

In general, limitations in CF muscle function have been linked to pulmonary disease and poor nutritional status (i.e. decreased muscle mass) [13]. Additional factors such as steroid use and inflammation may also play a role by decreasing muscle strength and power [14, 15]. Thus, reduced muscle function in CF may be the indirect result of CF disease or therapy. It has also been suggested that, in CF, the muscle is primarily affected by the disease as Cystic Fibrosis Transmembrane Conductance Regulator (CFTR) is expressed in healthy skeletal muscle [16]. In fact, in a mouse model, reduced contractile function of diaphragmatic muscle from CF-mice was observed in the presence of an inflammatory stimulus [17]. This might point to a qualitative problem of muscle function (i.e. intrinsic muscle dysfunction in CF). Indeed, the origins of reduced muscle function as well as the postulation of an intrinsic muscle dysfunction in CF are still a matter of debate [18, 19].

One important confounder in the assessment of muscle function and exercise capacity is the adjustment of the test results for variations in body and muscle size. This is of utmost importance, since reported differences in muscle power between people with CF and healthy controls might merely be the effect of inadequate scaling. In CF, body and muscle size are on average smaller than in healthy people. Only few studies have adequately adjusted for differences in body size when muscle function was evaluated. In most studies, either no adjustment was employed at all or a ratio to body weight was calculated, which has been proven to be an inadequate approach [20]. In the past, some studies in patients with CF have been conducted comparing data related to relative force level to avoid scaling based on body weight. In studies focusing on exercise data in children, allometric scaling has been used to address changes in body weight and height due to growth and maturation [21]. Decorte et al. related exercise results to muscle size, showing that differences between CF and control disappeared by doing so [22]. We think that controlling for muscle size is the most accurate approach to assess muscle function during exercise.

Besides muscle function, muscle metabolism is of interest when analysing aerobic and anaerobic exercise capacity. High-energy phosphate magnetic resonance spectroscopy has emerged as a non-invasive diagnostic tool to directly measure muscle metabolism [23]. This non-invasive technique has been proven feasible and has repeatedly been used in subjects with CF [6, 24, 25]. A couple of studies reported less muscular acidosis in patients with CF during exercise [2, 6, 25], which might point to a mitochondrial defect resulting in an altered cellular metabolism [26]. Other studies, though, could not confirm any differences in metabolic parameters in patients with CF compared to healthy controls [22, 27, 28].

The objective of this study was to assess muscle function in CF compared to healthy controls using adequate scaling methodology. Furthermore, we intended to analyse muscle metabolism using MRI spectroscopy during comparable exercise intensity between the groups. Combining MRI muscle spectroscopy with aerobic and anaerobic exercise will allow us to differentiate between a qualitative and a quantitative problem of muscle function and will enable us to relate assessment of local muscle power and metabolism to whole body exercise (i.e. Wingate test). Our hypothesis was that muscle function and metabolism would not be significantly different between CF and controls when controlled for differences in muscle size and that appropriate scaling will help clarify the question of a possible intrinsic muscle dysfunction in CF.

## Methods

### Population

Twenty patients with CF (CF, 6 female) and 23 age-matched healthy controls (CON, 10 female) participated in the study, which was approved by the local ethics committee (Ethics votum number: 9/05). Patients with CF were recruited from the local CF clinic, healthy controls were friends of the patients or hospital staff and their friends. The diagnosis of CF was proven by genetic testing and two separate pathological sweat tests [29]. Participants were aged 12 to 42 years and came for two study visits to the clinic.

### Lung function and exercise testing

After written informed consent was obtained, anthropometric data was collected and the participants performed a lung function test, where FEV_1_%predicted, FVC%predicted and RV/TLC were determined (Masterscreen Body, Jaeger, Würzburg, Germany) [30]. Further, diffusion capacity for carbon monoxide (TLCOC) was measured according to current standards [31].

Afterwards, participants were familiarized with the equipment and the upcoming tasks were explained: after taking place on the bike, patients pedalled for about 20 s without load to control adjustment of the saddle. During the establishment of monitoring (ECG cables (custocard m, Ottobrunn, Germany) and oxygen saturation (Nellcor Reflectance oxygen sensor RS10, Nellcor Puritan Bennet Inc., Pleasanton, CA, USA)) patients again received explanations on the upcoming tasks. Participants performed a Wingate anaerobic test [32] over 30 s and, after a break of at least 30 min, an incremental cardiopulmonary exercise test on a cycle ergometer (Ergomedic 834 E, Monark, Sweden) up to volitional fatigue according to the Godfrey protocol [33] while measuring gas exchange breath-by-breath (CPX/D, MedGraphics, St. Paul, MN, USA). Initial work load was 15 W in patients smaller than 150 cm and 20 W in patients taller than 150 cm; every minute, the load was increased by 15 W or 20 W, respectively. During exercise, participants were asked to maintain a cadence of 60 rounds per minute. The effort of the incremental cardiopulmonary cycling test was considered maximal if RER was > 1.03 [34] and the investigator had the impression of maximal exertion [35].

### MRI spectroscopy

**Fig. 1:**
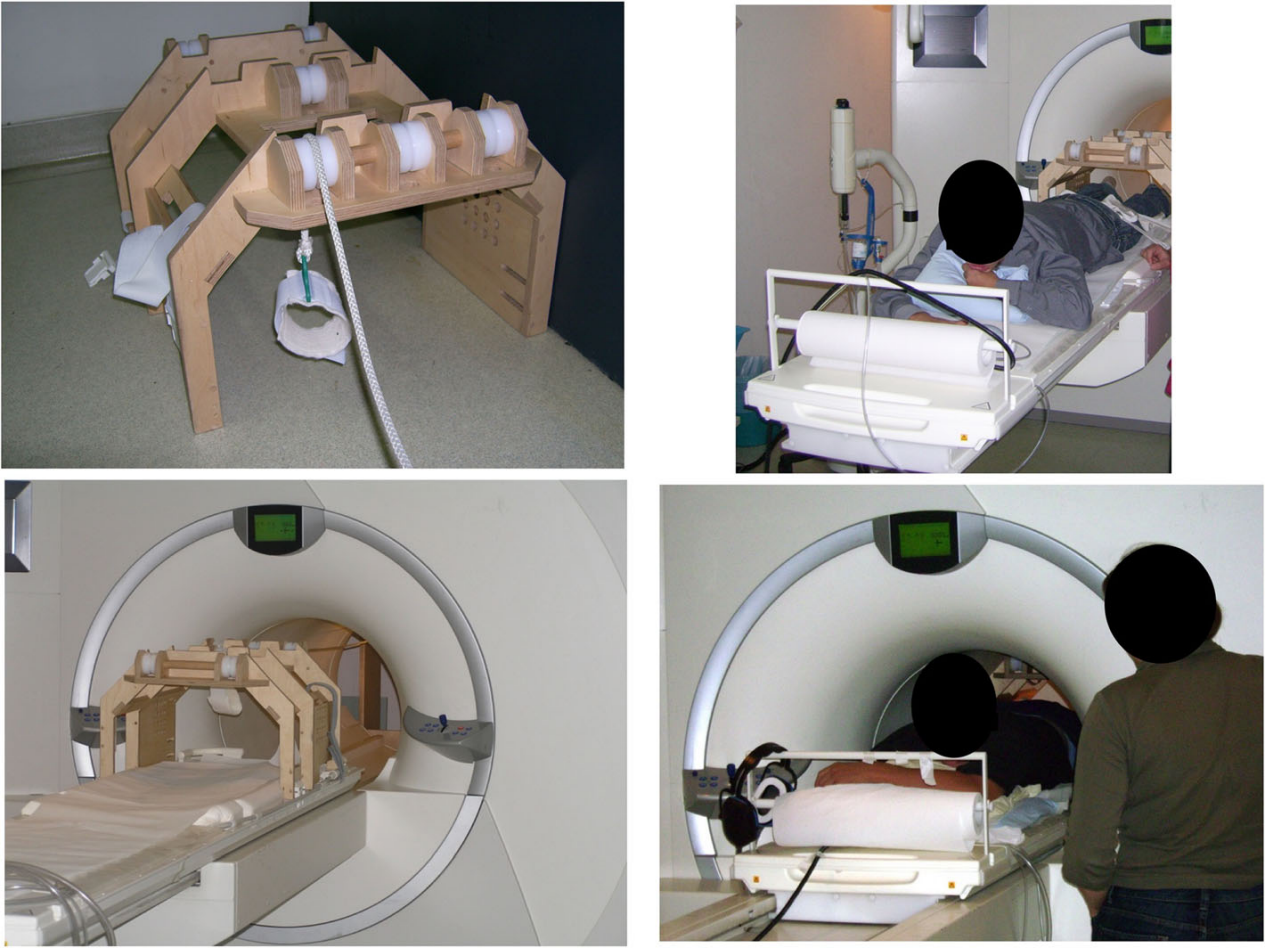
Self-built MRI compatible ergometer. Participants lay in prone position inside the MRI scanner. The ergometer was self-built and nonmagnetic (mainly built of wood). Moving of the work load was achieved via a pulley system. The left foot was secured to a padded foot loop. This loop was connected to a basket using a rope. Knee-extension led to an upward movement of the load. To ensure the correct placement of the thigh muscles on the magnetic coil, the thigh was secured to the coil using Velcro straps

On another day, with at least 2 days of rest between visits, participants underwent magnetic resonance spectroscopy at rest and during knee-extension exercises at increasing intensities up to volitional fatigue. Furthermore, muscle cross-sectional area of the *M. quadriceps* (qCSA) was assessed. The ergometer for the MRI was self-built and MRI-compatible; patients were in prone position and were asked to extend the knee of the left leg against increasing loads (see Fig. 1). Before starting the test in the MRI, equipment was demonstrated and the task explained. Participants lay in prone position, the leg was positioned on the coil and fastened with the help of Velcro straps. Then, participants performed 5 repetitions without load to get to know the exact task. This also served to ensure that the leg was securely positioned to prevent displacement from the coil during exercise. The test started with 8 min of rest for baseline measurements, followed by a steady state exercise over 5 min. Participants fully extended their knee against a workload every 2 s (i.e.30/min); an acoustic metronome helped keeping the rhythm. The workload for males was 60 g per kg body weight, for females 45 g per kg body weight, which was estimated to equal about 50–60% of maximal load. Maximal work load was assumed to be 0.12 per kg body weight in males and 0.09 per kg body weight in females. This exercise task was followed by 5 min of rest. Thereafter, participants performed an incremental exercise task with 5-min-stages starting with the same load as in the previous constant load task. 5-min-stages were chosen to achieve a steady state during each stage and to gather enough data to average good quality spectroscopy data. This approach with rather long stages has previously been successfully used in MRI spectroscopy [36]. Every 5 min 0.5–1 kg (depending on the patients’ anticipated maximal load) were added to the workload until volitional fatigue was reached. Depending on the anticipated maximal work load, the load of increments was chosen to reach the anticipated maximal load after an exercise time of about 45 min to generate comparable stages between participants. The achieved workload was defined as maximal workload (LastMRTmax). The test was followed by a 5-min-recovery period.

^31^P magnetic resonance spectroscopy was performed using a clinical 1.5 Tesla scanner (Magnetom Symphony Quantum, Siemens Healthcare, Erlangen, Germany). To assess the area under the peak for inorganic phosphate (Pi) and phosphocreatinine (PCr) the jmrui software package was used [37, 38]. Intracellular pH was calculated through chemical shift imaging (CSI) by recording localized voxel (Voxelsize 16 ml (2x2x4 cm)) with a 28 cm surface coil [39]. In the centre of the coil, the M. vastus intermedius of the left leg was positioned. Measurement time was 60 s for each spectrum (average of 30 spectra), all measurements were done timed to the leg extension cycle with full leg extension. Spectra were assessed at the end of the rest period, at the end of the initial constant load task, at the end of each 5-min exercise bout and at the end of the 5-min recovery period [36]. Next to rest, peak and recovery results, data during submaximal effort was analysed by averaging the values at 40–49%, 50–59%, 60–69%, 70–79%, 80–89% and 90–99% of maximal work load.

The incremental knee-extension test performed in the MRI was done with relatively low frequency of extensions and long stages. Maximal achievement may have been limited by maximal muscle force and not muscle metabolism. Therefore, a high-intensity high-frequency steady state knee-extension task was added in a subgroup of participants. After the above-mentioned incremental protocol, patients rested for 8 min. Then, they performed knee-extension exercises as described above but with a higher frequency (one repetition per second) and with a workload that was set to the individual maximal workload achieved in the incremental test plus an added kilogram for each participant. The test lasted until exhaustion which occurred within 2 to 3 min in all participants.

Changes in pH and Pi/PCR from baseline to peak exercise were calculated through chemical shift imaging as explained above [39].

### Statistical analysis

Patients’ characteristics are expressed as means and standard deviations. All data included in the linear models was normally distributed. Differences between CF and CON were calculated by using Student’s t-test. For correlation analyses, Pearson correlation coefficients were computed. Influence of CF-status, *M. quadriceps* cross-sectional area, and height on measures of performance were calculated using ANCOVA. In these models, dependent variables used were parameters of the Wingate test (peak power, mean power and power drop) and the maximal work load of the incremental knee-extension exercise in the MRI scanner. Due to the small sample size, especially in the subgroup performing the high-intensity exercise, models were calculated with a maximum of 3 covariates. With regard to the incremental knee extension task, pH and Pi/PCr were compared at rest, peak exercise and recovery and, as described above, at the averaged intervals at 40–49%, 50–59%, 60–69%, 70–79%, 80–89% and 90–99% of maximal work load in all participants. In the subgroup performing the high-intensity knee extension task, pH and Pi/PCr of this high-intensity task were compared at rest, peak exercise and recovery.

Further, pairwise t-tests were performed to compare pH and Pi/PCr at peak exercise between both knee-extension tests in the subgroup to analyse if the high-intensity protocol actually leads to a greater exertion. For statistical analyses, SPSS 22 (IBM) was used. Significant differences were assumed for *p* < 0.05.

## Results

Patients with CF were significantly smaller and lighter than healthy controls (Table 1). No significant difference was found in qCSA between groups. Lung function was worse in patients with CF reflected by a lower FEV_1_ (%predicted), a lower FVC (%predicted) and a higher residual volume/total lung capacity ratio (RV/TLC% predicted), diffusion capacity was comparable between the groups (Table 1). During the maximal incremental cardiopulmonary cycling test, patients with CF achieved a lower peak oxygen uptake (VO_2_max%predicted) than healthy controls despite a comparable effort as indicated by a similar respiratory exchange ratio (Table 1). This significant difference persists when controlled for qCSA (see Table 1).

**Table 1:** Participants’ characteristics/results of lung function and exercise testing

	Patients with CF (*n* = 20)	Healthy controls (*n* = 23)
Female (n/[%])	6 [30]	10 [43]
Anthropometric data		
Age (years)	21.7 ± 8 (12–42)	21.2 ± 6 (15–43)
Height (cm)	164.4 ± 12.0 (138–185)	174.5 ± 7.5 (160–192) ^***^
Weight (kg)	56.6 ± 13.9 (31–79)	67.8 ± 8.7 (52–94) ^***^
Lung function		
FEV_1_ (%pred)	74.3 ± 20.4 (25.7–103.0)	103.3 ± 12.2 (77.2–123.6) ^***^
FVC (%pred)	91.2 ± 17.0 (41.4–96.4)	101.0 ± 10.3 (77.2–125-8) ^*^
RV%TLC	30.7 ± 13 (23.7–41.8)	22.9 ± 4 ^*^ (19.3–31.3)
TLCO%	86.7 ± 17.4 (47.8–112.5)	94.4 ± 17.6 (67.8–128.0)
Incremental cycling exercise test (Godfrey protocol)		
VO_2_ peak (%pred)	77.8 ± 16.2 (44.3–110.1)	100.6 ± 18.8 (60.1–136-0) ^***^
VO_2_peak/qCSA ml/min	36.5 ± 7.4 (23.4–53.1)	49.1 ± 11.4 (23.5–77.7) ^***^
RER	1.17 ± 0.1 (0.9–1.4)	1.21 ± 0.1 (1.1–1.4)
Peak heart rate (bpm)	171 ± 15.4 (140–201)	183 ± 9.8 (166–202) ^**^
SPO_2_ peak exercise (%)	96 ± 2.6 (90–100)	98 ± 2.1 (91–100) ^*^

All parameters are reported as mean ± standard deviation (range); differences between participants with and without CF were calculated using Student’s t-test. Significant data is marked by ^***^p < 0.001, ^**^ *p* < 0.01, ^*^ *p* < 0.05

Characteristics of the subgroup performing the high-intensity, high frequency protocol are summed up in Table 4. Comparable to the whole cohort, patients with CF showed an impaired lung function and a reduced peak oxygen uptake. When comparing the subgroup of participants performing the high-intensity exercise task to the rest of the groups, a significant difference was evident for peak oxygen uptake in the control group (*p* < 0.001). Those performing the high-intensity knee-extension task showed a higher VO_2_ peak%predicted compared to the rest of the cohort (118% vs. 94%). No differences were found in the CF group between the subgroup and the total cohort.

### Muscle function

**Fig. 2:**
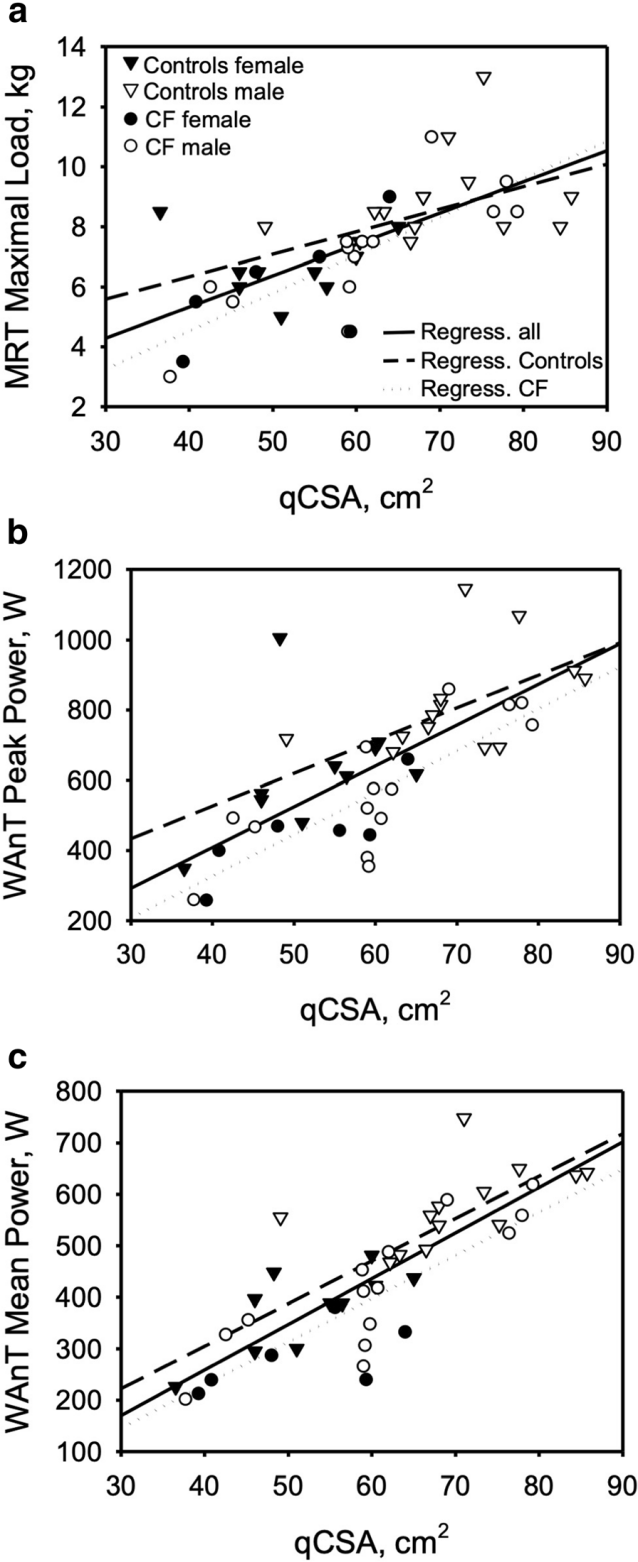
correlations between muscle cross sectional area (qCSA) and results of the MRI maximal workload (LastmaxMRT) and the Wingate anaerobic test. Regression lines are for the total cohort and separate for CF and CON (see legend of **a**). **a** correlation between qCSA and LastmaxMRT. **b** correlation between qCSA and peak power. **c** correlation between qCSA and mean power

Patients with CF showed a poorer performance in the Wingate anaerobic test reaching lower absolute peak power and mean power as well as a higher power drop (Table 2). However, when adjusting performance for height and qCSA using a linear model, there was no difference between groups in peak power, mean power nor power drop (Table 3). Similarly, a significant difference between groups was found in maximal work load of the incremental knee-extension test performed in the MRI (Table 2). Again, after adjusting for height and qCSA, disease status had no significant impact on the maximal load reached (see Table 3). Further, no differences were found in time to exhaustion between CF and control in the incremental knee-extension test, nor in the average weight of increments. Correlations of qCSA and outcome of the Wingate test and incremental knee-extension test are presented in Fig. 2, reflecting significant moderate to high correlation coefficients for qCSA and peak power (*r* = 0.713), mean power (*r* = 0.816) and maximal work load of the incremental MRI task (*r* = 0.676). In the subgroup, duration of the high-intensity exercise task showed no significant differences between controls and patients with CF (Table 4). Similarly, after adjusting workload for height and qCSA, participants of both groups achieved comparable results.

**Table 2:** Results of muscle function and muscle metabolism assessment

	Patients with CF (*n* = 20)	Healthy controls (*n* = 23)
Wingate anaerobic test		
Peak power (Watt)	537 ± 180 (258–860)	727 ± 186 (349–1145) ^***^
Mean power (Watt)	378 ± 127 (202–619)	486 ± 126 (226–747) ^***^
Power drop (Watt)	12 ± 5 (5–25)	8 ± 4 (2–15) ^***^
MRI spectroscopy – incremental knee-extension protocol		
Maximal load (kg)	6.8 ± 2.0 (3–11)	8.1 ± 2.0 (5–13) ^*^
Cross-sectional area *M. quadriceps* (cm^3^)	57.7 ± 12.4 (38–79)	62.8 ± 12.7 (27–86)
pH rest	7.1 ± 0.02 (7.04–7.31)	7.1 ± 0.02 (7.04–7.31)
pH maximum load	7.08 ± 0.06 (6.99–7.21)	7.08 ± 0.03 (7.02–7.19)
pH recovery	7.03 ± 0.04 (6.92–7.07)	7.04 ± 0.03 (6.97–7.11)
Pi/PCr rest	0.15 ± 0.03 (0.11–0.23)	0.15 ± 0.03 (0.11–0.21)
Pi/PCr maximum load	0.34 ± 0.09 (0.21–0.59)	0.36 ± 0.10 (0.23–0.63)
Pi/PCr recovery	0.14 ± 0.03 (0.09–0.18)	0.13 ± 0.02 (0.10–0.17)
Time to exhaustion (min)	41.2 ± 7.8 (27–64)	47.8 ± 8.4 (34–52)
Mean increment (kg)	0.8 ± 0.3 (0.5–1)	0.8 ± 0.2 (0.5–1)

All parameters are reported as mean ± standard deviation (range); differences between participants with and without CF were calculated using Student’s t-test. Significant data is marked by ^***^p < 0.001, ^*^ *p* < 0.05

**Table 3:** ANCOVA analysing the performance of Wingate anaerobic and incremental knee-extension tests during MRI spectroscopy adjusting for qCSA and height

	df	Significancy (p)	partial Eta-squared
Wingate test peak power (W)			
Model	3	< 0.000	.704
CF/non-CF	1	0.230	.125
Cross-sectional area M. quadriceps (cm^3^)	1	< 0.000	.301
Height (cm)	1	0.005	.188
Wingate test mean power (W)			
Model	3	< 0.000	.800
CF/non-CF	1	0.900	.072
Cross-sectional area M. quadriceps (cm^3^)	1	< 0.000	.510
Height (cm)	1	0.001	.246
Wingate test power drop (%)			
Model	3	0.001	.361
CF/non-CF	1	0.230	.037
Cross-sectional area M. quadriceps (cm^3^)	1	0.027	.119
Height (cm)	1	0.252	.034
Maximal work load during incremental test in MRI (kg)			
Model	3	< 0.000	.600
CF/non-CF	1	0.078	.002
Cross-sectional area M. quadriceps (cm^3^)	1	< 0.005	.183
Height	1	0.001	.247

Results of general linear models with Wingate test performance and maximal work load during MRI spectroscopy as dependent variable and disease status, height and cross sectional area of M. quadriceps as independent variables in the total cohort (*n* = 43). Effects of the respective factors on the model are presented as partial eta squared

**Table 4:** Participants’ characteristics/results of lung function, exercise testing, muscle function and muscle metabolism assessment in a subgroup performing a high-intensity, high-frequency constant load test to fatigue

Subgroup	Patients with CF *n* = 5	Healthy controls *n* = 10
Female (n/[%])	1 [20%]	6 [60%]
Anthropometric data		
Age (years)	23.6 ± 10.1 (13–40)	25.5 ± 4.0 (23–35)
Height (cm)	170.0 ± 14.1 (153–185)	173.5 ± 8.6 (160–187)
Weight (kg)	56.5 ± 15.4 (36.0–78.8)	67.8 ± 12.1 (52.0–94.0)
Cross-sectional area M. quadriceps (cm^3^)	59.4 ± 7.5 (48–69)	58.1 ± 10.1 (46–71)
Lung function		
FEV_1_%pred	74.0 ± 13.2 (58.8–90.1)	101.4 ± 17.3 (77.2–123.6) ^**^
FVC%pred	86.9 ± 11.0 (75.4–101)	98.3 ± 14.0 (77.2–111-9)
Incremental exercise test (Godfrey)		
VO_2_ peak (%pred)	77.2 ± 9.5 (87.4–102.1)	117.7 ± 11.1 (106.0–136-0) ^***^
Wingate anaerobic test		
Peak power (Watt)	560 ± 182 (379–8599	714 ± 215 (480–1145)
Mean power (Watt)	408 ± 136 (266–589)	465 ± 153 (295–748)
Power drop (Watt)	12.2 ± 4.4 (5.3–14.4)	8.4 ± 3.5 (7.6–20.6)
MRI spectroscopy - high-intensity knee-extension protocol		
Maximal load (kg)	7.9 ± 2.0 (4.5–11)	9.1 ± 2.5 (5–11)
Exercise time (sec)	135 ± 78 (99–156)	150 ± 51 (98–172)
Pi/PCr rest	0.16 ± 0.04 (0.09–0.16)	0.14 ± 0.03 (0.10–0.16)
Pi/PCr maximum	0.85 ± 0.45 (0.58–1.64)	0.96 ± 0.36 (0.42–1.44)
Pi/PCr recovery	0.15 ± 0.09 (0.09–0.13)	0.12 ± 0.06 (0.03–0.22)
pH rest	7.02 ± 0.02 (7.00–7.05)	7.03 ± 0.03 (6.99–7.02)
pH maximum	7.01 ± 0.05 (6.98–7.11)	7.01 ± 0.15 (6.81–7.25)
pH recovery	6.91 ± 0.17 (6.61–7.06)	6.96 ± 0.10 (6.82–7.11)

All parameters are reported as mean ± standard deviation; differences between participants with and without CF were calculated using Student’s t-test. Significant data is marked by ^***^p < 0.001, ^**^ *p* < 0.01

### Muscle metabolism

**Fig. 3:**
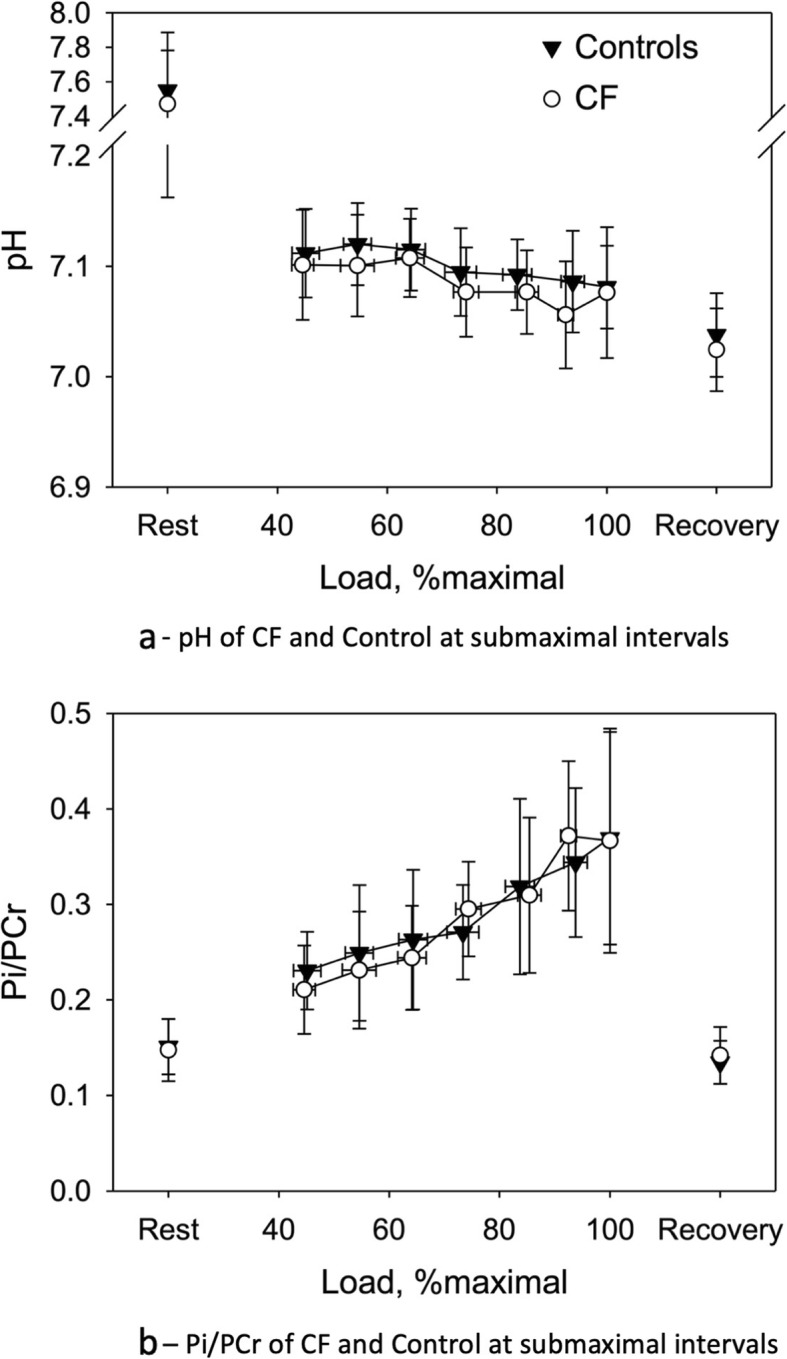
**a** pH of CF and Control at submaximal intervals – Data was averaged for the following ranges: 40–49%, 50–59%, 60–69%, 70–79%, 80–89% and 90–99% of maximal exercise. Data displayed are mean ± standard deviation. **b** Pi/PCr of CF and Control at submaximal intervals – Data was averaged for the following ranges: 40–49%, 50–59%, 60–69%, 70–79%, 80–89% and 90–99% of maximal exercise. Data displayed are mean ± standard deviation

In MRI spectroscopy, no differences were found between the groups in pH and Pi/PCr at rest, at peak exercise and after 5 min of recovery. Further, muscle metabolism was analysed during submaximal effort. Data at 40–49%, 50–59%, 60–69%, 70–79%, 80–89% and 90–99% of maximal exercise was averaged and compared between the groups. Again, no significant differences were present. See Fig. 3a and b for a graphic display of these results.

In the subgroup that performed the high-intensity, high frequency steady state knee-extension task, CF and CON also showed similar metabolic responses to exercise. Comparing metabolic results (pH and Pi/PCr at peak exercise) of the incremental with the high-intensity exercise with those of the incremental knee-extension task in the whole subgroup showed that pH as well as Pi/PCr were significantly different (*p* = 0.015 for pH and *p* < 0.001 for Pi/PCr) reflecting a greater muscle exertion during the high-intensity exercise.

## Discussion

In our study, muscle power was comparable between patients with CF and healthy controls when adjusted for muscle size. Further, we did not find evidence for intrinsic muscle dysfunction in CF while analysing muscle metabolism during exercise.

As expected, healthy controls showed a higher peak oxygen uptake during the incremental cardiopulmonary cycling test compared to patients with CF. This finding is in line with previous publications demonstrating a decreased aerobic physical fitness in CF [40]. After adjusting peak oxygen uptake as measure of aerobic performance to muscle size, differences between controls and patients with CF still remained significant. It has been well established, that VO_2_peak is associated with pulmonary function in CF [10] and impaired aerobic exercise capacity in CF has been largely attributed to a decreased pulmonary function [13] but changes in muscle function have also been discussed [2, 25, 28]. In order to focus on muscle function in more detail, Wingate testing and the above described tests in the MRI were chosen. Next to a whole-body exercise (i.e. Wingate test) testing muscle power, a localized muscle function test (i.e. incremental exercise test in the MRI) was performed in combination with the investigation of muscle metabolism. This enabled us to not only confirm the test results by two different exercise tasks but also to transfer these results to a whole-body exercise.

In our study, patients with CF scored significantly lower on the Wingate anaerobic test than healthy controls when looking at absolute values. This finding has been repeatedly reported [5, 6]. However, after adjustments for height and quadriceps cross sectional area (which may be regarded as surrogate parameters for muscle size), performance was comparable between patients with CF and healthy controls. As in the Wingate test, performance in the maximal knee-extension task during MRI spectroscopy was comparable between participants with CF and healthy controls after adjusting for differences in qCSA and height. Thus, lower muscle power of patients with CF observed in both different exercise tasks could be fully attributed to smaller muscle size; no functional differences to the leg muscles of healthy controls were observed. Therefore, the combination of both tests further emphasizes the importance of adjustment to body/muscle size in both, local muscle as well as whole body exercises. This finding is in line with another study showing that decreased absolute muscle power in patients with CF was explained by lower muscle mass [41]. In a further study examining muscle contractility and fatigability of the quadriceps muscle in 15 adults with CF, no significant differences compared to healthy controls were observed: the authors describe a trend for reduced muscle strength in CF which disappeared when adjusted for muscle cross-sectional area [28]. Various explanations for reduced anaerobic capacity in CF have been proposed in the past such as nutritional deficits, chronic systemic inflammation, corticosteroid therapy, and physical inactivity [10, 15, 42, 43]. A study with COPD patients showed that chronic hypoxia shifted the muscle fibre pattern towards type II fibres and lead to muscle atrophy [44]. Our study further emphasizes the important aspect of adjustment when it comes to comparing results of exercise testing and muscle performance. As mentioned in the introduction, adjusting to body weight has been challenged in the past [20]. In our study, differences in muscle size solely explained differences of muscle function tests between groups. Besides multiple tests of muscle function, the strength of our study is that we were able to adjust the results of muscle performance to muscle size.

Apart from the above-mentioned influences on muscle function, a primary defect of CF muscle has been discussed. CFTR is expressed in human muscle cells [16], which may result in altered muscle metabolism. In our cohort, no differences in muscle metabolism were detected between patients with CF and healthy controls in both, the incremental and the constant load high-intensity knee-extension tasks at rest, submaximal and peak exercise, and recovery. All changes of pH and Pi/PCr we measured during exercise are comparable to those reported in healthy children [45]. Our results on muscle metabolism in CF are in contrast to some previous studies: In one of these studies, patients with CF showed significantly less cellular acidosis and less changes in the Pi/PCr ratio during exercise compared with healthy controls [6]. However, work rate at peak exercise was not reported in this study. It is therefore unclear whether differences in muscle metabolism observed might be merely due to differences in exercise intensity between the groups. Another study assessed muscle metabolism in patients with CF and controls in a 30-s, 90-s and 5-min exercise task [25]. Only in the 90 s exercise bout less muscular acidosis was observed in patients with CF, while no significant difference in the Pi/PCr ratio was detected. During the shorter and the longer exercise bouts though, no significant changes in either pH or the Pi/PCr ratio were discovered [25].

In line with our results and contradictory to the above-mentioned studies, two further studies could not confirm impaired skeletal muscle oxidative metabolism in CF. In 10 adolescents with CF with normal lung function, no differences in muscle metabolism during an incremental exercise test were seen compared to healthy controls in MRI spectroscopy and near infrared spectroscopy [27]. This finding was confirmed by Decorte et al. when assessing muscle metabolism of the calf muscles in 15 adults with CF in comparison to healthy controls [22].

In the past, studies have assessed either muscle function or muscle metabolism in CF. The major strength of this study is that we simultaneously assessed muscle function and muscle metabolism by incremental and high-intensity constant-load exercise tests while using ^31^P magnetic resonance spectroscopy and combining this with the Wingate anaerobic test. Since our participants all performed at similar exercise levels during the spectroscopy, data on muscle metabolism can be validly compared at submaximal stages and at peak exercise. This approach strengthens the finding of a comparable muscle metabolism during dynamic high-intensity exercise in CF and healthy controls. In summary, we could show that differences in Wingate anaerobic test, incremental knee-extension protocol and high-intensity, high-frequency protocol could all be attributed to differences in muscle size (qCSA and height as surrogate markers) in ANCOVA analysis.

A limitation of our study is the rather small sample size and the fact that participants were age- but not gender-matched. In the subgroup performing the high intensity knee extension test, only 20% of participants were female, which may lead to delusive results as besides muscle mass endurance and fatigability may be different between men and women. Further, we did not assess regular exercise training of the participants which may have influenced exercise test outcome parameters.

## Conclusions

In summary, muscle function is comparable between patients with CF and healthy controls once differences in muscle size are accounted for. Further, there was no evidence for an intrinsic muscle dysfunction in patients with CF. More research is needed that covers larger cohorts, balances gender and can control for genotype and also disease severity to trust these results completely and gain further insight into potentially interrelated disease specific factors which may modulate muscle function in patients with CF. Since muscle power primarily seems to relate to muscle size, gaining muscle size may be a worthwhile approach to increase muscle power in this population. Besides optimizing nutrition, structured training interventions may become the focus of research to improve muscle power and thereby quality of life and maybe even disease severity.

## Data Availability

The datasets used for the current study are available from the corresponding author on reasonable request.

## Abbreviations

CF: Cystic fibrosis
CFTR: Cystic fibrosis transmembrane conductance regulator
CON: Control group
df: Degrees of freedom
FEV_1_: Forced expiratory volume in 1 s
FVC: Forced vital capacity
LastMRTmax: Maximal workload achieved during MRI task
MRI: Magnet resonance imaging
PCr: Phosphocreatine
Pi: Inorganic phosphate
qCSA: Quadriceps cross sectional area
RER: Respiratory exchange ratio
RV/TLC: Residual volume/total lung capacity
TLCO: Diffusion capacity for carbon monoxide
VO_2_peak: Peak oxygen uptake
WAnT: Wingate Anaerobic test
